# Editorial: Reading acquisition of Chinese as a second/foreign language, volume II

**DOI:** 10.3389/fpsyg.2023.1292975

**Published:** 2023-09-27

**Authors:** Linjun Zhang, Zaizhu Han, Yang Zhang

**Affiliations:** ^1^School of Chinese as a Second Language, Peking University, Beijing, China; ^2^State Key Laboratory of Cognitive Neuroscience and Learning, Beijing Normal University, Beijing, China; ^3^Department of Speech-Language-Hearing Sciences, Masonic Institute for the Developing Brain, University of Minnesota, Minneapolis, MN, United States

**Keywords:** reading acquisition, Chinese as a second/foreign language, phonological awareness, morphological awareness, cognitive factors

There has been a significant increase in the number of Chinese as a Second/Foreign Language (CSL/CFL) learners worldwide, particularly over the past two decades, due to a combination of economic, cultural, educational, and technological factors. However, learning Mandarin Chinese presents unique challenges for second language learners as they face specific hurdles such as tone acquisition and the complexity of Chinese morphology and writing (Kecskés and Sun, 2017; Gong et al., 2020; Ke, 2020; Wen, 2020; Chan et al., 2022). “*Reading Acquisition of Chinese as a Second/Foreign Language (CSL/CFL) – Volume II*” features a collection of 11 articles that represent continuing efforts within a general behavioral and neurocognitive framework to provide informative and insightful original findings on the Research Topic (Zhang et al., 2023).

Two child-focused studies addressed early learning challenges. Chan et al. investigated Chinese character acquisition in culturally diverse second language (L2) learners, comparing 491 L2 children (aged 3 to 6) to 240 first language (L1) peers in Hong Kong kindergartens using the Chinese Character Acquisition Assessment (Chan et al., 2020). The data revealed not only L2 learners' strength in sound and meaning but also struggles with character forms, emphasizing the importance of early oral support for L2 preschoolers. Zhou and McBride examined the connection between Cantonese phonological awareness, Mandarin Pinyin invented spelling, and English spelling in multilingual first and second language Cantonese-speaking second and third graders in Hong Kong. While both groups demonstrated similar skills in Cantonese phonological awareness and Mandarin Pinyin invented spelling, L1 speakers outperformed L2 speakers in Mandarin Pinyin tone skills, suggesting phonological transfer effects in multilingual contexts.

Three studies examined cross-language influences in adult learners. Hu and Zhao investigated code-switching costs in Chinese–English relative clause processing. The results highlighted the contribution of syntactic processing, particularly in head movement during relative clause comprehension, to these costs. Yan et al. examined how bilingualism and English proficiency may influence the processing of the Chinese sentence-final particle “le.” Participants included English-dominant second language learners (L2), heritage learners, and monolingual Mandarin speakers. The study found that heritage learners showed sensitivity to semantic conditions similar to the target language, influenced by early exposure and positive cross-linguistic influence from English. This indicates that early bilingualism can shape the processing of Chinese sentence structures in heritage learners. Chai and Bao explored the influence of linguistic differences between learners' native languages (L1) and Chinese on character, vocabulary, and grammar acquisition. Using data from 58,240 Chinese as a second language (CSL) learners with diverse L1 backgrounds and proficiency levels, the study employs the World Atlas of Language Structures (WALS) index to measure linguistic distance. Results show that closer linguistic distance aids character and vocabulary acquisition across proficiency levels, but as proficiency increases, linguistic distance hinders grammar acquisition, indicating proficiency-dependent effects on CSL learners.

Three studies delve into cognitive factors in L2 Chinese reading. Yang et al. assessed 252 international students from Pakistan, Indonesia, Malaysia, and Laos and revealed that linguistic and cognitive skills account for 80% of L2 Chinese reading variation, with a significant role played by morphological awareness. Path analysis underscores the influence of these skills on reading comprehension through higher-order cognitive skills, highlighting the significance of morphological awareness in L2 Chinese reading. Feng and Jiang explored the context predictability effect (Li et al., 2022) and prediction error cost in reading, highlighting challenges and slower reading in advanced Chinese L2 learners during predictive processing. Wang et al. studied the impact of metacognition monitoring on L2 Chinese audiovisual comprehension. Findings suggest that absolute calibration accuracy significantly predicts L2 Chinese audiovisual comprehension, while relative calibration accuracy does not, emphasizing the importance of considering various aspects of metacognition monitoring in L2 learning strategies and addressing individual learner differences.

Three studies used electrophysiogical and neuroimaging methods. Liu et al. investigated how native Mandarin speakers and L2 learners process quadrisyllabic idiomatic expressions (QIEs) in Chinese. L2 learners processed high-frequency QIEs faster, while native speakers handle low-frequency ones more quickly, possibly due to semantic satiation. The fMRI data showed native speakers used the anterior cingulate for cognitive control during high- and low-frequency QIE comparisons. For comparing idiomatic and pseudo-idiomatic constructions, semantic processing in bilateral temporal poles dominated, suggesting that native speakers rely on conceptual understanding, rather than syntax, when processing Chinese idiomatic expressions. Ding et al. studied how conceptual concreteness may influence the acquisition and integration of novel words into semantic memory via thematic relations. Abstract words were harder to acquire and recall, but both concrete and abstract words integrated into semantic memory, as indicated by behavioral and ERP measures in a lexical decision task. Chen et al. investigated how Mandarin Chinese native speakers and highly proficient second language (L2) learners from South Korea process complex sentences with center-embedded relative clauses. Results showed both groups exhibited a consistent biphasic ERP waveform pattern, indicating interactive syntactic and semantic processing in Chinese. This challenges the idea of temporal and functional priority in syntactic processing found in morphologically rich languages and highlights cross-linguistic consistency.

These studies collectively contribute to a better understanding of the challenges and factors influencing reading acquisition for Chinese as a second language. They highlight the role of early reading challenges, cross-linguistic transfer, metacognition monitoring, idiomatic expression processing, thematic relations and semantic memory, linguistic distance, code-switching costs, sentence structure processing, context predictability, and the impact of heritage language exposure in the development of reading skills among L2 learners of Chinese. The findings range from uncovering neural and cognitive mechanisms to informing language teaching practices, calling for further research in this field into the intricacies of language learning, cross-linguistic transfer, and neurocognitive processes in diverse linguistic contexts.

## Author contributions

LZ: Writing—original draft. ZH: Writing—original draft. YZ: Writing—review and editing.
